# New directions in migraine

**DOI:** 10.1186/1741-7015-9-116

**Published:** 2011-10-25

**Authors:** Greg A Weir, M Zameel Cader

**Affiliations:** 1 Medical Research Council Functional Genomics Unit, Department of Physiology, Anatomy and Genetics, University of Oxford, Oxford, UK

## Abstract

Migraine is a highly prevalent neurological disorder imparting a major burden on health care around the world. The primary pathology may be a state of hyperresponsiveness of the nervous system, but the molecular mechanisms are yet to be fully elucidated. We could now be at a watershed moment in this respect, as the genetic loci associated with typical forms of migraine are being revealed. The genetic discoveries are the latest step in the evolution of our understanding of migraine, which was initially considered a cerebrovascular condition, then a neuroinflammatory process and now primarily a neurogenic disorder. Indeed, the genetic findings, which have revealed ion channels and transporter mutations as causative of migraine, are a powerful argument for the neurogenic basis of migraine. Modulations of ion channels leading to amelioration of the migraine 'hyperresponsive' brain represent attractive targets for drug discovery. There lies ahead an exciting and rapidly progressing phase of migraine translational research, and in this review we highlight recent genetic findings and consider how these may affect the future of migraine neurobiology and therapy.

## Introduction

Migraine is a common, episodic neurological disorder characterised by severe headaches, and in about one-third of cases it is preceded by a focal, transient neurological phenomenon termed the 'aura'. Whether migraine with aura (MA) and migraine without aura (MO) are distinct or overlapping entities is somewhat contested, but it is not uncommon for patients to experience both forms of migraine. Migraine affects up to 18% of women and 6% of men, with a peak incidence between 25 and 55 years of age [1]. The disease has long fascinated clinicians and scientists, with descriptions of migraine-like symptoms appearing in antiquity; however, only recently have the pathogenic mechanisms started to be unravelled. This is particularly true for MA, which is perhaps a more homogeneous and tractable condition for study than MO, because the phenotype of MA is well defined and genetics likely play a greater role [2].

The migraine aura is likely caused by 'cortical spreading depression' (CSD), a wave of intense neuronal and glial depolarization followed by a period of inactivity, slowly progressing over the cortex [3,4]. The trigeminal system (TGVS), consisting of the meningeal and superficial cortical blood vessels that are innervated by the trigeminal nerve, is strongly implicated in the initiation of the headache pain [5]. The TGVS projects to the trigeminal nucleus caudalis (TNC) in the brainstem, which in turn projects to higher-order pain centres. Animal models provide evidence that CSD could be linked to activation of the TGVS, thus providing a hypothetical triggering mechanism for the migraine headache [6]. However, such a link in the human brain is still controversial [7], and although silent CSD has been speculated to be a cause, the relationship of CSD and MO is uncertain.

In this review, we consider the emerging genetic discoveries about migraine, beginning with familial hemiplegic migraine, where the search for causative genes has been particularly rewarding. The more typical forms of migraine have been considerably more challenging, but we describe the exciting discoveries of the past year. The discovery of a role for the TWIK-related spinal cord potassium channel (TRESK) in migraine, in particular resurrects the debate over the relative importance of peripheral and central mechanisms in migraine pain, and the origin of the headache is therefore considered. We next highlight the growing importance of glial cells in migraine pathogenesis, as evidenced by the expression pattern of migraine-causative genes, and finally we describe potential directions for migraine treatment.

## Familial hemiplegic migraine

Genetic factors are clearly involved in the aetiology of migraine, as illustrated by twin studies [8]. Complex segregation analysis has illustrated a non-Mendelian inheritance pattern [9], revealing the multigenic nature of migraine. Often a profitable approach to studying such complex genetic diseases is to interrogate an autosomal dominant subtype of the condition. Familial hemiplegic migraine (FHM) is a rare, monogenic subtype of MA [10]. Clinically, FHM patients experience a headache phase similar to that experienced by common migraineurs, but FHM is distinguished by additional prolonged hemiparesis. To date, three genes, when disrupted, have been identified as causing FHM: *CACNA1A *(FHM1) [11], encoding the α1 subunit of neuronal Ca_v_2.1 Ca^2+ ^channels; *ATP1A2 *(FHM2) [12], encoding the α2 subunit of Na^+^/K^+ ^ATPase pumps; and *SCNA1 *(FHM3) [13], encoding the pore-forming α1 subunit of neuronal Na_v_1.1 Na^+ ^channels. More recently, a mutation in *SLC4A4 *[14] encoding the Na^+^-HCO_3_^- ^cotransporter NBCe1 has been identified in sisters with FHM, and a mutation in *SLC1A3 *[15] encoding the glial glutamate transporter EAAT1 has been identified in one patient with pure hemiplegic migraine.

**Figure 1:**
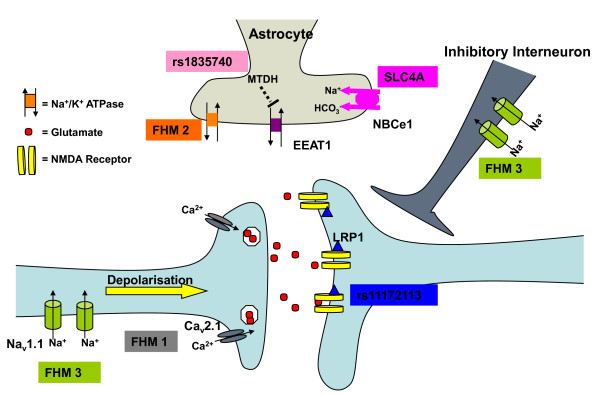
**Migraine mutations affecting the central glutamate synapse**. Increased Ca2+ influx caused by familial hemiplegic migraine subtype 1 (FHM1) associated mutations in Cav2.1 channels enhance glutamate release from presynaptic terminals. Loss of Na+/K+ ATPase function, as seen in FHM2, indirectly reduces astrocyte uptake of glutamate, resulting in increased levels of the neurotransmitter in the synaptic cleft. FHM3 associated mutations can reduce firing of inhibitory interneurons or potentiate presynaptic action potential generation. Mutations in *SLC4A4 *inhibit glia-mediated acid secretion and thus free *N*-methyl-D-aspartate (NMDA) receptors from proton-mediated inhibition. Activity of EAAT1, the major glutamate transporter in the brain, is directly affected by a mutation in its sequence and indirectly by upregulation of *MTDH*, a likely consequence of a reported mutation in rs1835740. *LRP1 *has a role in glutamate signalling and has been shown to directly modulate NMDA-dependent calcium currents *in vitro*.

The common thread between these genes adds weight to the notion that FHM (and hence potentially common migraine) is essentially a channelopathy [16] (Figure 1). Cellular and knockin mouse models of FHM1 have provided evidence for a gain-of-function effect of *CACNA1A *mutations on channel function [11,17]. Ca_v_2.1 channels are expressed presynaptically by neurons and couple depolarisation of the membrane with neurotransmitter release [18]; thus a channel 'gain-of-function' would predict increased neurotransmission [19]. A more complex spectrum of Na_v_1.1 defects, including loss and gain of function, can cause FHM3 [20]. Data derived from a Na_v_1.1-knockout mouse model show that a loss of one allele predominantly decreased activity of GABAergic inhibitory interneurons whilst having no effects on excitatory pyramidal neurons [21]. In contrast, mice with overexpression of a gain-of-function Na_v_1.2 channel mutation, causing a seizure phenotype, chiefly activated excitatory hippocampal pyramidal neurons [22]. These findings demonstrate well that the final effect on central nervous system (CNS) excitability is very dependent on whether the predominant effect of a mutation is on inhibitory or excitatory neurons. The importance of glia is evident in FHM2, and all studied mutations yield a 'loss-of-function' of the Na^+^/K^+ ^ATPase pump [23]. These pumps are essential for the uptake and clearance of neurotransmitters and K^+ ^ions from the synaptic cleft by astrocytes, so loss of function mutations could result in an increase in extracellular neurotransmitter and K^+ ^levels. Neuronal excitation causes extracellular alkalosis, but this is buffered by acid secretion from glial cells. Such secretion is induced by glial cell depolarization and mediated by inward rectifying Na^+ ^HCO_3_^- ^cotransport [24]. Mutations in *SLC4A4*, another glial transporter that has been reported to cause FHM in two sisters, cause defective trafficking of NBCe1 and reduced intracellular alkalization in C6 glioma cells [14]. Decreased capacities to buffer extracellular alkalosis could result in increased neuronal excitability, as *N*-methyl-D-aspartate (NMDA) receptors are sensitive to inhibition by protons [24].

While FHM and migraine overlap significantly, phenotypic differences do exist [25]. Despite this, FHM serves as a highly valuable model of migraine. The usefulness of FHM genetic studies extends beyond simply identifying genes responsible for rare migraine subtypes. More importantly, they have provided mechanistic insights into the pathobiology of migraine in general, which seems likely to be a disease related to ionic disturbances with a resultant altered excitability of certain areas of the brain. Adopting this hypothesis may help prioritize findings among the large volume of data arising from genome-wide association studies and genome sequencing by examining ionic homeostasis pathways.

## Migraine

Although multiple genes have been implicated in FHM, evidence for genetic alterations associated with common forms of migraine had been lacking. There have been numerous linkage studies, and despite some replication, the genetic abnormalities at these loci remain unknown [26,27]. Very many association studies of candidate genes have also been undertaken but have been hampered by the relatively small sample sizes. Since 2010, several genetic loci have been identified in association with 'typical' migraine. The first was found in a large, genome-wide association study comprising 2,731 MA patients at three separate European headache clinics. The minor allele of rs1835740 on chromosome 8q22.1 was shown to be linked to migraine with and without aura [28]. Two genes, *MTDH *and *PGCP*, flank the region. More functional work is needed to determine the relevance of such polymorphisms to *MTDH *and *PGCP *gene expression; however, it is interesting to note that both genes have a role in regulating levels of extracellular glutamate in the brain, a neurotransmitter heavily implicated in migraine pathogenesis [29]. A second study published in 2011 identified three additional susceptibility loci for common migraine [30]. The three SNPs mapped within or near transcribed regions of known genes: *LRP1*, *PRDM16 *and *TRPM8*. *LRP1 *is highly expressed by neurons and is found in association with NMDA receptors in dendritic synapses [31] linking the SNP with disrupted central glutamate signalling. While the plausible link between *PRDM16 *and migraine is not obvious, the association of *TRPM8 *is intriguing. The gene encodes for a sensor of cold and cold-induced burning pain, which is primarily expressed in sensory neurons such as the trigeminal ganglion and the dorsal root ganglion [32]. *TRPM8 *is considered to have a role in animal models of neuropathic pain [33], a disorder that shares some similarities with migraine [34].

The genome-wide association studies in migraine have yielded plausible susceptibility loci, but further work is still required to confirm that the identified SNPs are causally related and the adjacent genes are relevant to migraine. An alternative approach that can yield more immediate insights into disease pathogenesis is family-based genetic studies. A candidate gene approach followed up by linkage and functional analysis revealed a mutation in *KCNK18*, the gene encoding TRESK, that segregated with MA in a large pedigree. TRESK is a member of the two-pore potassium channel family showing neuronal expression [35]. *KCNK18 *was chosen for screening, as the channel was known to play a prominent role in neuronal excitability and therefore was postulated to participate in pain pathways [36]. Furthermore, volatile anaesthetics such as halothane have been shown to activate TRESK and are known to inhibit CSD [37]. One migraine proband was identified as carrying a two-bp deletion in the *KCNK18 *gene, which leads to premature truncation of the protein. The frameshift mutation F139WfsX24 was found to cosegregate perfectly with the migraine phenotype in relatives of the proband. Patch-clamp recordings from *Xenopus *oocytes revealed a loss of protein function and a dominant-negative action of mutant subunits on wild-type TRESK channels. Further work confirmed that TRESK is expressed in migraine-salient areas such as the trigeminal ganglion, the cortex and the dorsal root ganglion [38]. In a mouse knockout model, loss of TRESK results in a 20% increase in sensitivity to thermal pain [39]. It is expected that a loss of K^+ ^leak current would lead to the reduced rheobase current required for neuronal spiking, as shown in dorsal root ganglion neurons of TRESK^-/- ^mice [40]. The TRESK mutation was identified in only one large family, and therefore further studies are required to confirm any broader role in migraine pathogenesis. Nevertheless, given the strong expression and physiological function of TRESK in trigeminal ganglia and the central role of the trigeminal ganglia in migraine, modulation of TRESK is a viable target for migraine therapy. Furthermore, the protein has no close paralogues, and known compounds have already been shown to influence channel activity [21].

## Origin of the headache

**Figure 2:**
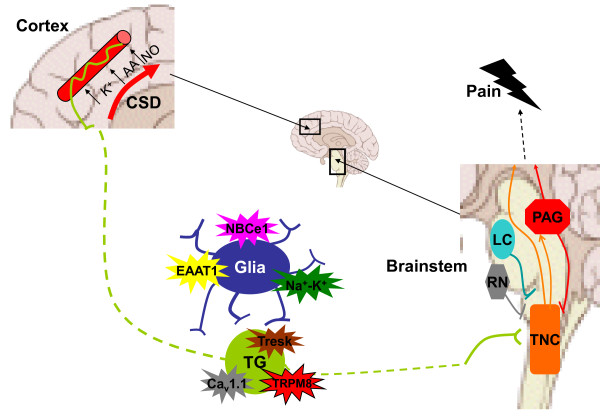
**Neuronal circuitry in migraine pain generation**. Cortical spreading depression (CSD) triggers plasma protein extravasation from dural blood vessels, which in turn activates trigeminal (TG) afferents. Multiple mutations have been found in familial and sporadic migraine that could reduce the threshold for firing of TG neurons, either directly by affecting neuronal excitability or indirectly by modulating local glia activity. Signals are transduced to the trigeminal nucleus caudalis (TNC), which receives several modulatory inputs from other areas of the brainstem, such as the periaqueductal gray (PAG), the locus coeruleus (LC) and the raphe nuclei (RN). These areas have been proposed as sites of dysfunction in migraine. The TNC projects to rostral brain areas, where the perception of pain is generated.

While CSD is largely accepted as the neurophysiological correlate of the aura, the cause of the headache phase of migraine is less established and there is considerable debate whether the pain is peripheral or central in origin. Early observations by Harold Wolff [41] that the superficial temporal artery becomes dilated during migraine and that electrical stimulation of dural and cerebral arteries results in nausea and the perception of pain strikingly similar to that in migraine [42] led to the idea that the vasculature has a role to play in headache initiation. Whilst cerebral blood flow changes are clearly not the primary pathology in migraine, the trigeminovascular system is still considered by many to be the essential substrate of migraine pain (Figure 2). Indeed, in rodents, CSD has been shown to cause activation of the trigeminovascular system along with dilatation of the middle meningeal artery, both of which are abolished by ipsilateral trigeminal denervation [6]. Proponents of an intrinsic CNS mechanism point instead to dysfunction of the brainstem as the principal source of the headache. Neuromodulatory structures such as the periaqueductal gray (PAG), the locus coeruleus (LC) and the raphe nuclei (RN) modulate responses to afferent traffic [5]. Positron emission tomography has revealed activation of such areas of the brainstem during acute attacks. Additionally electrical stimulation of the PAG can induce migraine-like headache pain [43]. The relative importance of central and peripheral structures in migraine headache will become apparent as the expression pattern of migraine genes is characterized. For example, the identification of *KCNK18 *and *TRPM8 *as migraine-associated genes, both highly expressed in sensory neurons, would certainly support the importance of peripheral neurons. This in turn has implications for drug design and delivery, since blood-brain barrier permeability may not be a prerequisite for a migraine headache therapeutic drug.

## Neuron and glia interactions

The multigenic nature of migraine lends itself to the idea that multiple perturbations in homeostasis are required for disease pathology. Evidence derived from FHM mutations points to an important role of astrocytes in migraine. During late gestation and the early neonatal stage, *ATP1A2 *is expressed predominantly in neurons; however, by adulthood, the gene is expressed primarily in astrocytes. This expression switch may explain the clinical phenotype switch seen in patients. Young FHM2 patients often have infantile epilepsy (presumably as a consequence of neuronal hyperexcitability), whereas by adulthood the symptoms are more of the classical FHM2 type, that is, migraine attacks caused by impaired neurotransmitter uptake by astrocytes [44].

Astrocytes are critical in modulating the neuronal microenvironment, as they control extracellular ionic composition and prevent accumulation of neurotransmitters in the synaptic cleft [45]. Spinal glial activation has been suggested to play a role in a variety of pain states [46]. Intracellular Ca^2+ oscillation in ^cultured ^astrocytes ^shows significant similarity to CSD [47] and has been suggested to contribute to the propagation of spreading depression [48]. However, the role of glial calcium waves *in vivo*, where intracellular Ca^2+ oscillation ^has yet to be robustly shown, is contested [49]. The release of proinflammatory mediators by glia may also contribute to central sensitisation of migraine and to the development of symptoms such as allodynia [32,50]. Chronic opioid treatment can result in hyperalgesia and allodynia, potentially by activating glial cells [51], whilst administration of naloxone, which blocks microglial activation, enhances the analgesic effect of morphine [52].

It is therefore becoming increasingly clear that glia have a role beyond simply myelination and maintaining neuronal homeostasis. They appear to directly and actively influence the excitability of neurons and as such may represent a novel means of modulating neuronal activity and responsiveness.

## Migraine therapy

Therapies are broadly divided into acute symptomatic and prophylactic. To date, triptans are the gold standard in acute migraine therapy. They are potent 5-hydroxytryptamine (serotonin) subtypes 1B and 1D receptor agonists that, when delivered orally, are effective in 29% to 64% of patients, depending on the criteria used to define pain relief: (1) pain-free after two hours and (2) moderate or severe pain to mild or no pain after two hours, respectively [53,54]. However, clinical use can be limited by their potent vasoconstriction [55]. The most significant recent advance in acute treatments relates to calcitonin gene-related peptide (CGRP) receptor antagonists. Stimulation of TG neurons results in the release of CGRP [56]; high levels of CGRP are found in external jugular venous blood during migraine attacks [57]; and infusion of CGRP can cause MO [58]. Telcagepant (the first orally administered CGRP antagonist) has been shown to possess efficacy similar to that of zolmitriptan as acute therapy [59]. It also shows improved safety and tolerability [60], although it caused elevated levels of liver transaminases in a minority of patients taking the drug twice daily for three months [61]. Insights gleaned from genetic studies offer novel drug targets and potential prophylactic agents through alterations in neuronal excitability. Indeed, current prophylactics such as valproate, verapamil, topiramate and lamotrigine are known to have effects on migraine-associated ion channels [62,63]. Several drugs that block glial cell activation are also available and have approved clinical safety profiles. These drugs include naltrexone, naloxone, minocycline and ibudilast, and some have already been shown to be effective as prophylactics for migraine [24,64,65].

## Summary and future directions

Migraine is a multigenic condition in which aberrant ion channels are becoming increasingly implicated in pathogenesis as inducing an altered state of peripheral and central neuronal excitability. It is realistic to anticipate that further genetic studies may reveal other tractable drug targets, and this will help unravel the pathogenic process in migraine, which is essential for the rational development of effective treatments. Although each protein may be genetically relevant to only a small subset of migraineurs, each one reveals important information about migraine mechanisms potentially applicable to the wider migraine population. It is likely that therapies must be targeted against general pathways involved in neuronal excitability, which will mean addressing both neuronal and glial activation. Furthermore, it may be crucial to understand the interplay of neurons and glia, given that at least two FHM genes are of key importance for glial function. Indeed, simply targeting the neuronal ion channels may be either partially or completely ineffective if glial adaptive changes circumvent any therapeutic intervention. The use of triptans (and soon likely CGRP antagonists) has made major leaps forward in patient care, but for a multigenic, complex condition such as migraine, it may be that only therapies targeting multiple pathways and cell types will lead to drugs with broader and more sustained efficacy. Equally true is that a better understanding of the genetic architecture of migraine with the future availability of rapid whole-genome sequencing in an individual could lead to the ever sought after personalized medicine.

## Abbreviations

bp: base pair; SNP: single-nucleotide polymorphism.

## Competing interests

The authors declare that they have no competing interests.

## Authors' contributions

GAW drafted the manuscript. MZC critically revised the manuscript and gave final approval for its publication.

## Pre-publication history

The pre-publication history for this paper can be accessed here:

http://www.biomedcentral.com/1741-7015/9/116/prepub
